# Promising Perspectives of the Antiproliferative GPER Inverse Agonist ERα17p in Breast Cancer

**DOI:** 10.3390/cells12040653

**Published:** 2023-02-18

**Authors:** Marilena Kampa, Rosamaria Lappano, Fedora Grande, Bruno Rizzuti, Marcello Maggiolini, Elias Castanas, Yves Jacquot

**Affiliations:** 1 Laboratory of Experimental Endocrinology, School of Medicine, University of Crete, 71003 Heraklion, Greece; 2 Department of Pharmacy, Health and Nutritional Sciences, University of Calabria, 87036 Rende, Italy; 3 CNR-NANOTEC, SS Rende, Department of Physics, University of Calabria, 87036 Rende, Italy; 4 Institute of Biocomputation and Physics of Complex Systems, Joint Unit GBsC-CSIC-BIFI, University of Zaragoza, 50018 Zaragoza, Spain; 5 CiTCoM, CNRS UMR 8038, INSERM U1268, Faculty of Pharmacy of Paris, University Paris Cité, CEDEX 06, 75270 Paris, France

**Keywords:** apoptosis, GPER, peptide, triple-negative breast cancer

## Abstract

The estrogen receptor α (ERα) corresponds to a large platform in charge of the recruitment of a panel of molecules, including steroids and related heterocyclic derivatives, oligonucleotides, peptides and proteins. Its 295–311 region is particularly targeted by post-translational modifications, suggesting that it could be crucial for the control of transcription. In addition to anionic phospholipids, the ERα 295–311 fragment interacts with Ca^2+^-calmodulin, the heat shock protein 70 (Hsp70), ERα and possibly importins. More recently, we have demonstrated that it is prone to interacting with the G-protein-coupled estrogen receptor (GPER). In light of these observations, the pharmacological profile of the corresponding peptide, namely ERα17p, has been explored in breast cancer cells. Remarkably, it exerts apoptosis through GPER and induces a significant decrease (more than 50%) of the size of triple-negative breast tumor xenografts in mice. Herein, we highlight not only the promising therapeutic perspectives in the use of the first peptidic GPER modulator ERα17p, but also the opportunity to modulate GPER for clinical purposes.

## 1. Introduction

The 66 kDa human estrogen receptor α (ERα), which belongs not only to the nuclear steroid receptor superfamily but also to transcription factors, binds a panel of molecules with diverse chemical structures. A number of small molecules (typically with MW < 650 g/mol) endowed with estrogenic activity, such as di- and tri-arylethylenes, phenolic stilbenes, coumestans, isoflavones and pollutants, interact within the same ~450 Å^3^ binding pocket as that occupied by the endogenous female hormone 17β-estradiol (E2).

Regarding genomic (direct) mechanisms, the agonist-bound conformation of ERα allows its phosphorylation and dimerization (homo- or heterodimerization, depending on the context), the recruitment of specific co-activators (through an LxxLL motif, where L corresponds to leucine and x to any other amino acid) and finally the association of the preformed complex with small DNA regions called estrogen response elements (EREs). This latter association, which involves the ERα DNA-binding domain, is partially directed by two zinc atoms tetrahedrally coordinated to four cysteines and forming two zinc fingers, i.e., the D and P boxes [1]. In this regard, it should be noted that divalent metal cations such as copper, cobalt or nickel have been reported to bind within the same pocket as E2 to activate gene transcription [2]. Depending on the post-translational changes and related secondary and tertiary structure modifications, ERα is also in charge of the recruitment of co-regulatory proteins participating in the allosteric modulation of the expression of genes and, therefore, of the biological response [3].

The non-genomic (indirect) mechanisms appear to be much more complex. They involve different types of estrogen receptors (ERα66, ERβ, GPER, ERα36, ERα46, etc.) that work in concert with growth factor receptors to activate specific protein kinases. Interestingly, the molecular mechanisms associated with non-genomic events are closely linked to the local flexibility of specific regions in the vicinity of the ligand-binding pocket of ERα which appear, therefore, as a paradigm for structural investigations aiming to better explain the biological relevance of the spatial dynamics of ERα.

**Figure 1:**
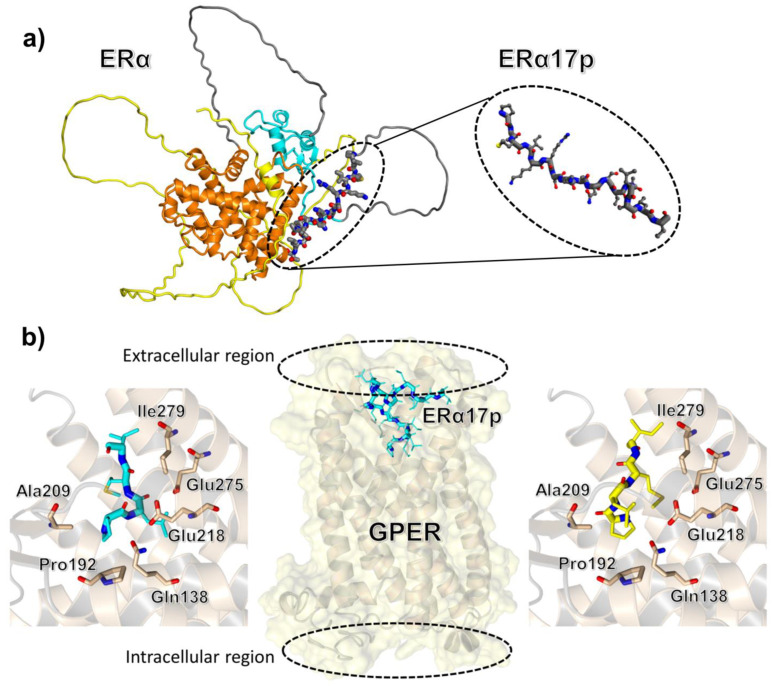
(**a**) Drawing of the 295–311 sequence of the peptide ERα17p, in the context of ERα. Due to the presence of several disordered regions, AlphaFold was used to predict the protein conformation [19] The structured domains overlap with those solved by crystallography and used in molecular modeling, confirming the relevance of our approach [20]. Domains are highlighted in different colors, including the N-terminal domain (NTD), residues 1–180 (yellow), DNA-binding domain (DBD), residues 181–253 (cyan), ligand-binding domain (LBD), and residues 302–552 (orange), except sequence 295–311, shown in detail (in grey). (**b**) Binding of the peptide ERα17p and its N-terminal PLMI motif to GPER. Center: ERα17p bound to a GPER structure model with the extracellular 50-residue disordered region of the receptor omitted. Left: Details of the N-terminal anchoring region PLMI of ERα17p predicted by molecular dynamics simulation. Right: Pose of the tetrapeptide PLMI predicted by molecular docking. In all cases, hydrogen atoms are omitted and side-chain bonds are represented by using smaller sticks compared to the peptide backbone.

The human ERα is composed of four distinct domains: (1) a A/B domain (residues 1 to 180), also called AF1 for ligand-independent transactivation function 1; (2) a C domain (residues 181 to 262), for the DNA-binding domain; (3) a D domain (residues 263 to 302), which corresponds to the hinge region; (4) an E/F domain (residues 303 to 595), which is defined as the ligand-dependent activation function AF2. The fragment defined by amino acids 295 to 311 (sequence: P^295^LMIKRSKKNSLALSLT^311^, Figure 1a) is issued from the hinge (residues 295 to 302) and AF2 (residues 303 to 311) regions and is strongly targeted by post-translational modifications such as methylation [4], acetylation and phosphorylation [5], ubiquitination [6] and SUMOylation [7]. The K^299^RSKK^303^ motif, which corresponds to the third nuclear localization signal (NLS) of ERα, is targeted by proteolytic enzymes [8,9]. This surface-exposed ERα region is principally folded into left-handed polyproline II (PPII) and overhangs a type II β-turn (amino acids Arg-363 to Asp-369) [10,11], two regular structures usually found in protein regions in charge of the recruitment of protein partners [12,13]. The peptide corresponding to the 363–369 β-turn interacts physically with the FK1 domain of the co-regulatory protein FKBP52 (for FK506-binding protein of 52 kDa) [14,15]. Moreover, its orientation depends on the pharmacological profile of the bound ligand (i.e., E2 versus diethylstilbestrol versus raloxifene versus tamoxifen) [10]. The deletion of the 295–311 fragment is responsible for constitutive transcription [16] and the mutation to arginine of the residue Lys-303 (K303R) confers resistance not only to tamoxifen but also to the aromatase inhibitor anastrozole [17]. Altogether, these observations strongly suggest that the 295–311 part of the autonomous AF2 domain (AF2a) is key for transcription [18]. In the light of the conformational, post-translational and binding characteristics of this region of ERα, exploring the effects of the peptide corresponding to the 295–311 17-mer sequence (i.e., ERα17p, Figure 1a) in different contexts may be particularly relevant for a better understanding of the physiological and pathological functions supported by ERα and E2.

## 2. The Peptide Corresponding to the ERα Residues 295–311 Is Responsible for Apoptosis

Under E2 treatment and by using mass spectrometry, we have observed that different fragments issued from the 295–311 sequence were produced in the extracellular space of hormone sensitive cells, after the proteasomal degradation of ERα. Considering that the 295–311 residues regulate transcription, we have hypothesized that the resulting fragments could act as a “relay” during the turnover of the receptor and that they could interfere with the fate of neighboring cancer cells through a paracrine mechanism, even in hormone refractory cells [21,22]. Even if their concentrations, when endogenously produced, are still unknown, we have studied the action of the parent peptide corresponding to the sequence 295–311 (ERα17p, sequence: H_2_N-PLMIKRSKKNSLALSLT-COOH) at the concentration of 10 μM, as it corresponds to the concentration required to reach optimal effects. Furthermore, ERα17p elicits cell growth and ERE-dependent gene transcription [16,23]. It interacts with Ca^2+^-calmodulin with a stoichiometry ratio of 2:1, suggesting that it may stabilize ERα dimers [24,25]. It interacts also with Hsp70 [26] and ERα itself, revealing that it could be involved in homodimerization [23]. In the same context and with recombinant ERα, ERα17p abrogates the recruitment of LxxLL coactivatory motifs [23]. It interferes also with the recruitment of the PPII motifs of the co-activators PNRC and PNRC2 [27]. More recently, a direct interaction with the G-protein-coupled estrogen receptor (GPER) has been proposed (Figure 1b and Table 1) [28]. Interestingly, it engulfs anionic vesicles and micelles, suggesting that the ERα 295–311 region could participate, in the context of the whole protein, in the stabilization of ERα in the cytoplasmic membrane (Table 1) [29,30]. A K_d_ value of 1.2 ± 0.3 μM was calculated with eukaryotic cell membrane models [30], giving weight to the biological relevance of this interaction. Thus, events occurring at the cell membrane could explain, at least in part, the mechanism of action of ERα17p.

In the light of previous results, we have explored the ability of ERα17p to bind cell membranes. An interaction was evidenced by confocal imaging microscopy and a FACS analysis by using an FITC-labeled version of ERα17p, in both ERα-positive and -negative breast cancer cells, suggesting an ERα-independent process [35]. Since ERα17p did not compete with the association of E2 in the cytoplasmic membrane but rather enhanced it, a form of interaction with a membrane estrogen site(s) differing from ERα was pointed out [35]. The experiments performed using tritiated or biotinylated ERα17p derivatives demonstrated that a small amount of peptide was internalized in cells within the first hour of incubation [29,36,37]. Even if doubts persisted concerning the involvement of a different form of membrane estrogen receptor or the translocation of the classical receptor ERα, subsequent evidences showed the coexistence of both mechanisms. Thus, an involvement of GPER was suspected in both steroid-deprived and complete serum conditions (see next section for more details).

In breast cancer cells, membrane-initiated E2 effects are known to prevent apoptosis. Based on the action of ERα17p on the apoptotic fate of breast cancer cells, its effects, alone or in combination with E2-BSA, were investigated [35]. In ERα-positive cells (T47D, MCF-7) and under serum-deprived conditions, which correspond to major pro-apoptotic conditions, ERα17p decreased apoptosis. In breast cancer cells not expressing ERα (SKBR3 and MDA-MB-231), ERα17p exerted apoptosis and reversed the anti-apoptotic action supported by E2-BSA. This was further confirmed by ERα17p’s effects in breast cancer cells and serum conditions. In such conditions, ERα17p rapidly induced (within the first 6 h of incubation) apoptosis in a time-dependent manner and in all tested cell lines, independently from the presence of ERα [35]. However, the ERα-positive cells were more sensitive to the presence of the peptide (apoptosis still observed at 12–24 h) than the negative ones, particularly in SKBR3, in which apoptosis faded after 12 h to afford massive necrosis [35]. In ERα-negative SKBR3 cells, which are considered as one of the most resistant breast cancer cell lines towards apoptosis, ERα17p induced apoptosis both in the presence and in the absence of serum [35]. Even though ERα17p displays apoptosis in breast cancer cells independently from ERα, it may have dual effects, depending not only on the presence of serum but also of ERα. Indeed, ERα could impact the duration of apoptosis and direct cells towards apoptosis or necrosis.

**Figure 2:**
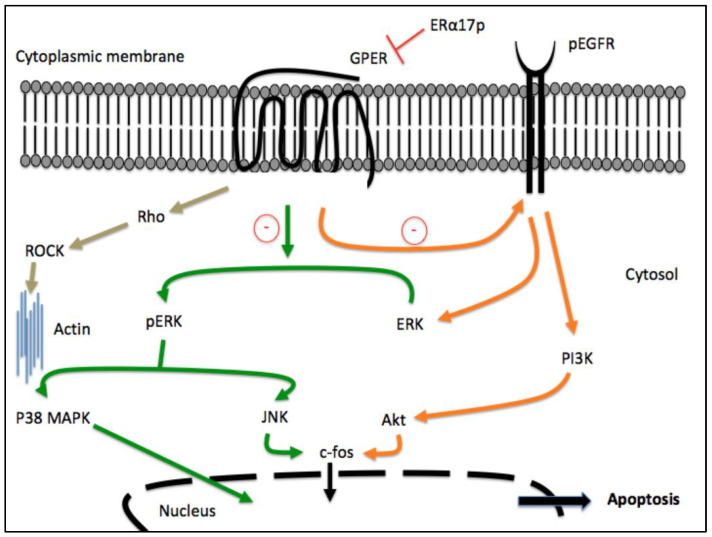
GPER-dependent apoptosis pathways induced by ERα17p in breast cancer cells. ERα17p may interact with the extracellular ligand-binding domain of GPER and induce its downregulation. Decreased levels of pEGFR and pERK, which are followed by the downregulation of the GPER target gene c-fos, are observed through a mechanism implying PI3/Akt, p38 MAPK and JNK transduction pathways. When ERα17p interacts with GPER, it can also inhibit or activate the Rho/Rock cascade, depending on the cell line, modifying actin polymerization and cell migration. Importantly, the proteins shown in this figure have been experimentally demonstrated to be involved in the mechanism of action of ERα17p.

Next, we deciphered the mechanism through which ERα17p is apoptotic in complete serum. We observed an alteration of the expression of Bcl2 family members, suggesting a mitochondria-related (intrinsic) mechanism [35]. The exposure of cells to ERα17p for 24 h and at the concentration of 10 μM induced a decrease in the Bcl-_xL_/Bax ratio and an increase in cleaved caspase-9 [35]. These effects were found to be mediated by specific intracellular signaling pathways primarily involving p38 MAPK and c-jun N-terminal kinases (JNK), as shown in Figure 2 [38]. In connection with apoptosis, ERα17p was also found to reduce the clonogenic survival and proliferation rate of breast cancer cells (T47D, MCF-7, SKBR3 and MDA-MB-231) [28,35].

The transcriptional data obtained from above breast cancer cell lines (i.e., T47D, MDA-MB-231 and SKBR3) support the pharmacological profile of ERα17p. In these three cell lines, ERα17p induces indeed massive early changes in gene transcription. ERα- and non-ERα-related signatures resulting from ERα17p treatment were therefore examined. The analysis of the genes modified by ERα17p showed ERα-related genes modified by E2 and involved not only in major cellular functions such as cell cycle, proliferation, apoptosis, inflammation and immune functions, but also in transport, signaling and nuclear processes [36]. Significant percentages (25 to 32%, depending on the cell line) of genes were modified by ERα17p but not by E2, suggesting that ERα17p exhibits a non-ERα-related signature [36]. The GSEA analysis of these non-ERα-related transcripts revealed genes involved in apoptosis, the actin cytoskeleton and cell migration [36]. Depending on the cell line and independently from ERα, ERα17p at 10 μM either inhibited (T47D and SKBR3 cells) or enhanced (MCF7 and MDA-MB-231 cells) cell migration, through specific intracellular signaling pathways implying the phosphatidylinositol-3 kinase (PI3K)/Akt (all cell lines), Rho/ROCK (T47D, MCF7 and MDA-MB-231) and p38 MAPK (SKBR3 cells), as shown in Figure 2 [38]. However, a concomitant action of the peptide through the intranuclear pool of ERα cannot be totally excluded, its size being compatible with a passive diffusion through nuclear pores. In this regard, we recently identified the third NLS of ERα (i.e., K^299^RSKK^303^ motif), which is present in ERα17p, as putatively targeting importin α [39]. Thus, ERα17p could represent a regulator for the translocation of ERα for its proper transcriptional activity or other nuclear processes. This hypothesis could explain, at least in part, the opposite effects displayed by ERα17p in steroid-deprived and complete serum conditions. In the same context and in serum-free conditions, ERα17p provokes in ELT3 Leiomyoma cells a delayed increase in the translocation of β-arrestin, a protein that contributes to multiple aspects of the downregulation, signaling and trafficking of GPCRs [37].

The pro-apoptotic action of ERα17p was further verified in vivo [35]. BalbC^−^/^−^ mice bearing xenografts of MDA-MB-231 cells were treated with ERα17p for four weeks with a dose of 1.5 mg/kg body weight, three times a week. Remarkably, ERα17p decreased by more than 50% the size of the aforementioned xenografts compared to the control. The histological analysis of the ERα17p-treated tumors showed increased apoptosis followed by massive central necrosis [35]. These results were in total agreement with the in vitro data. Finally, it should be stressed that Ki-67 immunostaining revealed a reduced proliferation rate of the cells at the periphery of the so-called “growing edge” of the tumor [35].

In summary, ERα17p exerts strong apoptotic or anti-proliferative effects through a specific transcriptional signature involving well-defined kinases (Figure 2). These effects are observed both in vitro and in vivo, with impressive tumor regression outcomes and without apparent toxicity for the liver or other organs [35]. The affinity of ERα17p for breast tumors could result not only from the high concentration of phosphatidylserine (an anionic lipid) in the inner leaflet of the membrane of cancer cells [40], but also from its specificity for mammary glands [28]. Even if the classical ERα is not a prerequisite for ERα17p apoptotic action, its presence could have some modulatory functions.

## 3. Participation of GPER in the Anti-Proliferative Action of ERα17p

During our investigations, we have shown that the selective GPER antagonist G-15 was able to reverse the migratory action of ERα17p [38]. Likewise, we have demonstrated that an anti-GPER siRNA was prone to abrogating the effects of ERα17p in ELT3 cells [37]. Thus, GPER seems to be required for the pharmacological activity of ERα17p. The protein GPER is a class A (rhodopsin-like) G-protein-coupled receptor (GPCR) that is localized to the cytoplasmic membrane, as well as to intracellular compartments such as the endoplasmic reticulum, the Golgi apparatus and even the nucleus, in some specific conditions [41,42]. This receptor attracted interest over the past years, as its ability to mediate estrogenic effects in both physiological and pathological processes, including breast cancer, has been reported [43,44,45,46]. Briefly, GPER signaling triggers the transactivation of the epidermal growth factor receptor (EGFR) through the matrix metalloproteinase (MMP)-mediated release of EGF-like ligands and the subsequent generation of transduction signals, including the activation of PI3K and mitogen-activated protein kinase (MAPK) pathways. Moreover, it increases cAMP concentrations and the mobilization of intracellular calcium [46,47]. Thereafter, it mediates the transcription of diverse genes, including the oncogene c-fos and the connective tissue growth factor (CTGF), which are typically used as molecular sensors of GPER action [48]. Many GPER-regulated genes are involved in the growth and progression of diverse tumors, such as breast cancer [49,50]. It is worth mentioning that in breast cancer, increased GPER levels have been associated with worse disease outcome features, such as an increased tumor size, distant metastases and tamoxifen resistance [51,52,53]. In line with these findings, bio-informatic analyses of data issued from large cohorts of patients have revealed that the expression of GPER was correlated with pro-metastatic genes in breast tumors lacking the classical ERα [54]. Notably, in breast cancer cells as well as in cancer-associated fibroblasts (CAFs), diverse stimuli including growth factors, hormones and hypoxia enhance GPER levels toward aggressive features of the tumor environment, such as cell proliferation, migration and angiogenesis [48,55]. On these bases, the expression of GPER may be not only related to the cancer cells’ sensitivity to estrogens and response to endocrine therapies, but also to the prediction of aggressive breast tumor phenotypes. As such, GPER may represent a promising therapeutic target for more comprehensive strategies to treat breast cancer and other types of malignancies [56].

GPCR-targeting peptides are emerging as promising therapeutics for the treatment of multiple diseases, as outlined by the approval of more than fifty of these molecules for clinical use, particularly for metabolic diseases or cancer, including breast tumors [57,58]. Most of the approved GPCR-targeting synthetic peptides function as agonists, and as such replace or enhance low levels of endogenous peptides. Few antagonists have been developed, whereas no peptidic inverse agonists or allosteric modulators, to our knowledge, have been reported to date for clinical purposes [58]. The use of antagonists or inverse agonists could represent the most intuitive strategy to interfere with GPCR signaling, as specific inverse agonists may, indeed, counteract either the ligand-dependent or -independent activation of a defined receptor. Such an approach should be considered for anti-cancer therapeutics, GPCRs being frequently overexpressed in specific cancer types besides being constitutively activated [59].

Considering that the overexpression of GPER is crucial in the progression of breast cancer, further studies (aside from those discussed in the previous paragraph) were carried out to investigate in greater detail the molecular mechanisms by which ERα17p may engage the GPER transduction pathway. In ERα-negative and GPER-positive SKBR3 breast cancer cells as well as in serum conditions, ERα17p decreases the basal (constitutive) activity of GPER, revealing an inverse agonist profile [28]. In similar conditions, it triggers the proteasome-dependent downregulation of GPER [28], a regulatory mechanism usually observed in the pharmacology of hormone receptors and preventing any overwhelming response [60]. The decrease in GPER levels led to reduced EGFR and ERK1/2 phosphorylation and c-fos expression towards anti-proliferative cell effects (Figure 2) [28]. The involvement of GPER in the anti-proliferative action of ERα17p was confirmed by a 50% decrease in the latter by G-36 [28]. Further observations demonstrated the localization of ERα17p within the plasma membranes together or not with GPER in diverse breast cancer cell lines [28,30,35]. Strikingly, experimental results have shown recently that the PLMI peptide, which corresponds to the N-terminus of ERα17p, was sufficient to closely mimic the anti-proliferative effects of the whole peptide [28,32]. This motif has also been claimed to direct the interaction between ERα17p and Ca^2+^-CaM [37] and to present the considerable advantage of not being amyloidogenic, in contrast with ERα17p, which exhibits a primary amphipathic character [32,33].

Overall, the aforementioned findings provide evidence regarding the inverse agonism exerted by ERα17p (and the PLMI motif) on GPER, even if further studies are required to confirm these data (for example by using a reconstituted GPER as a model system). Likewise, our panel of observations improves the physiological relevance of ERα17p with respect to GPER:

- In vitro and in vivo biological responses of ERα17p are modified by the GPER antagonists G15 or G36 [28,37,38,61] or by the GPER agonist G1 [28,37];
- ERα17p and GPER co-localize at the cytoplasmic membrane, as shown by using fluorescence microscopy, a fluorescent version of ERα17p and the anti-GPER antibody TA35133 [28];
- A GPER siRNA abrogates ERα17p’s effects [37];
- ERα17p is inactive in a GPER knockout (KO) cellular model obtained by CRISPR/Cas9 [32].

In this regard, it should be stressed that no effects are observed with a scramble peptide derived from ERα17p, confirming that the activity displayed by ERα17p is sequence-specific and that it occurs through a specific protein [28].

Docking and molecular dynamics (MD) simulation studies through a protocol similar to the one used to demonstrate the binding of other ligands to GPER confirmed the interaction of ERα17p in the GPER extracellular ligand-binding domain (Figure 1b) [28,62,63,64,65,66,67,68,69]. This interaction occurs with an affinity of −7.2 kcal/mol, which corresponds to a dissociation constant (K_d_) in the low micromolar range [28]. Due to the size of ERα17p, the C-terminal region of the peptide seems to compact at the entrance of the protein cavity [28]. This was observed in a molecular dynamics run performed in fully hydrated conditions and on a relatively long timescale (>10 ns). Strikingly and as suggested by previous biochemical studies, the association of ERα17p with GPER is mediated by the sole N-terminal tetrapeptide motif PLMI, which shares structural analogies with the GPER antagonist PBX1 [28,62]. More precisely, the N-terminal proline, which corresponds to the anchoring motif of ERα17p to GPER, forms a hydrogen bond with either the Gln-138 or the Ala-209, and hydrophobic contacts with the Pro-192, whereas the side chain of the C-terminal isoleucine points towards the Ile-279 (Figure 1b). Due to the dynamics of GPER, the different binding modes found for the PLMI sequence indicate that it can populate two slightly distinct conformations that may easily interconvert. Thus, both molecular docking and classical molecular dynamics simulations essentially agree with a single and well-defined bound conformation of the PLMI motif [28]. These observations are important not only because they corroborate with biology, but also because they provide some clear indications for the rational design of ERα17p analogues.

To summarize, the N-terminal region of ERα17p, and especially the starting proline residue, should be strictly conserved to preserve the binding properties of the whole peptide. These findings corroborate the fact that the sole N-terminal region of ERα17p deeply penetrates into the receptor to mediate the biological response. In light of our docking and experimental investigations, ERα17p should be considered as a peptidic GPER modulator, although further studies are warranted to provide direct evidence regarding its binding properties to GPER.

## 4. Conclusions

Our findings clearly suggest that peptides such as ERα17p deserve further investigation, especially in the context of the modulation of GPER and novel breast cancer therapy approaches. Interestingly, ERα17p shares not only anti-proliferative and apoptotic activities but also anti-inflammatory and anti-nociceptive actions through GPER, thereby opening new perspectives in the management of inflammatory breast cancer and tumor-mediated pain [52,61]. Its weak intracellular uptake and its propensity to bind anionic lipids could assist its direct interaction with GPER. Besides the therapeutic interest of ERα17p, the PLMIKRSKKNSLALSLT 17-mer sequence could constitute, in the context of ERα (residues 295–311) and ERα36 (residues 123–139), an interaction platform in charge of the recruitment of GPER [11]. Lastly, its N-terminal part should be considered as a hit for structure–activity relationship studies and the synthesis of new molecules with multimodal actions, through GPER.

## Figures and Tables

**Table 1:** List of the direct interactions in which the 295–311 region of ERα is involved.

**Interaction partners of the 295–311 region of ERα (in the context of the whole protein)**
Ca^2+^-calmodulin [16]
**Direct partners of the 295–311 region of ERα (in the context of the peptide ERα17p)**
Ca^2+^-calmodulin [16,24,25,31] ERα17p, to form amyloid fibrils, hydrogels and complex aggregates [30,32,33] Estrogen receptor α [21] GPER [28] Heat Shock Protein 70 (HSP70) [26] Hard and soft negative lipid-containing surfaces including cell membrane models [29,33,34]
